# Temporal Lobe Spikes Affect Distant Intrinsic Connectivity Networks

**DOI:** 10.3389/fneur.2021.746468

**Published:** 2021-12-17

**Authors:** Laura Mirandola, Daniela Ballotta, Francesca Talami, Giada Giovannini, Giacomo Pavesi, Anna Elisabetta Vaudano, Stefano Meletti

**Affiliations:** ^1^Department of Biomedical, Metabolic, and Neural Sciences, Center for Neuroscience and Neurotechnology, University of Modena and Reggio Emilia, Modena, Italy; ^2^Neurology Unit, “San Giovanni Bosco” Hospital, Torino, Italy; ^3^Neurology Unit, Azienda Ospedaliero-Universitaria of Modena, Ospedale Civile Baggiovara (OCB) Hospital, Modena, Italy; ^4^PhD Program in Clinical and Experimental Medicine, University of Modena and Reggio Emilia, Modena, Italy; ^5^Neurosurgery Unit, Azienda Ospedaliero-Universitaria of Modena, Ospedale Civile Baggiovara (OCB) Hospital, Modena, Italy

**Keywords:** EEG, fMRI, TLE, BOLD, epilepsy, temporal lobe, EEG-fMRI

## Abstract

**Objective:** To evaluate local and distant blood oxygen level dependent (BOLD) signal changes related to interictal epileptiform discharges (IED) in drug-resistant temporal lobe epilepsy (TLE).

**Methods:** Thirty-three TLE patients undergoing EEG–functional Magnetic Resonance Imaging (fMRI) as part of the presurgical workup were consecutively enrolled. First, a single-subject spike-related analysis was performed: (a) to verify the BOLD concordance with the presumed Epileptogenic Zone (EZ); and (b) to investigate the Intrinsic Connectivity Networks (ICN) involvement. Then, a group analysis was performed to search for common BOLD changes in TLE.

**Results:** Interictal epileptiform discharges were recorded in 25 patients and in 19 (58%), a BOLD response was obtained at the single-subject level. In 42% of the cases, BOLD changes were observed in the temporal lobe, although only one patient had a pure concordant finding, with a single fMRI cluster overlapping (and limited to) the EZ identified by anatomo-electro-clinical correlations. In the remaining 58% of the cases, BOLD responses were localized outside the temporal lobe and the presumed EZ. In every patient, with a spike-related fMRI map, at least one ICN appeared to be involved. Four main ICNs were preferentially involved, namely, motor, visual, auditory/motor speech, and the default mode network. At the single-subject level, EEG–fMRI proved to have high specificity (above 65%) in detecting engagement of an ICN and the corresponding ictal/postictal symptom, and good positive predictive value (above 67%) in all networks except the visual one. Finally, in the group analysis of BOLD changes related to IED revealed common activations at the right precentral gyrus, supplementary motor area, and middle cingulate gyrus.

**Significance:** Interictal temporal spikes affect several distant extra-temporal areas, and specifically the motor/premotor cortex. EEG–fMRI in patients with TLE eligible for surgery is recommended not for strictly localizing purposes rather it might be useful to investigate ICNs alterations at the single-subject level.

## Introduction

Simultaneous EEG and fMRI (EEG–fMRI) recording is a functional neuroimaging technique that reveals cerebral hemodynamic changes related to interictal epileptiform discharges (IED) visualized on scalp EEG. It combines the temporal resolution of the EEG with the spatial resolution of MRI, offering the opportunity to define epileptogenic foci or complex epileptic networks (1, 2). In the last 20 years, several EEG–fMRI studies have been performed in patients with epilepsy to better understand specific epileptic networks in focal and generalized epilepsies. The hemodynamic response to IED could result in BOLD increments (or fMRI activations), reflecting spike-generating fields or decrements (deactivations), which can be interpreted as areas of reduced neuronal activity, although the meaning of deactivation is still debated (3, 4). In focal epilepsies, studies have detected BOLD-signal changes that are tightly coupled with the regions generating focal IED and are concordant with intracranial EEG findings (5). Compared to intracerebral recordings that are targeted to few cerebral structures based on *a priori* hypothesis, the spatial resolution of EEG–fMRI allows covering the whole brain and can highlight the involvement of deep brain structures in seizure generation or propagation, and their relationships with the cortex (3). Patients with temporal lobe epilepsy (TLE) have previously been studied with EEG–fMRI (3, 6–11). Modifications of BOLD signal related to temporal IED were observed in 50–83% of the patients in the different studies, and often, but notably not only in all cases, the fMRI response was localized in the temporal lobe, ipsilateral, or/and contralateral to the IED but also in extra-temporal or subcortical structures. These findings are in line with the recent concept of “network epilepsy” applied to TLE, which has overcome the traditional hypothesis that in focal epilepsy seizure activity originates from a specific and anatomically isolated focus (12, 13). Ideally, EEG–fMRI could represent a non-invasive way to identify the seizure onset zone as part of the presurgical evaluation, but its clinical use is still limited. Modifications of the BOLD signal in relation to IED do not distinguish between irritative zone, seizure onset zone, or propagation effect (14). The main limitations of studies that focused on its clinical role in the presurgical evaluation were the small number of patients enrolled and the number and features of IED recorded during the scan. Efforts have been made to overcome the issue, and methods have been applied in patients without visually detectable IED on scalp EEG (15). A few retrospective EEG–fMRI studies on patients with TLE undergoing epilepsy surgery found that if the BOLD response was located in the area that was surgically removed (concordant fMRI response), the postsurgical outcome was better than if the BOLD response also involved regions outside the surgical resection (discordant fMRI response) (10, 16). Studies on group analysis of EEG–fMRI data aimed to reveal common network alterations in patients with TLE. BOLD activations related to IED were found not only in the ipsilateral mesial and neocortical temporal cortex, but also in the insula and the cerebellum, while deactivations were observed in structures belonging to the default mode network (DMN) (8, 14), a physiological cerebral network encompassing brain areas preferentially active during conscious rest, including the precuneus and posterior cingulate, bilateral temporo-parietal, and medial prefrontal cortices (17). Previous resting-state fMRI (rs-fMRI) studies in TLE patients showed alterations of the major intrinsic connectivity networks (ICN) (18). Mainly, the activity of the DMN, of attention, and of the executive control networks showed significant differences in patients with TLE when compared to healthy subjects.

In the present prospective study, 33 patients with TLE undergoing EEG–fMRI as part of their presurgical workup were consecutively enrolled at the University of Modena and Reggio Emilia—Epilepsy Center. We not only aimed to verify the epileptogenic zone (EZ) through this technique but also to evaluate the patient's specific epileptic network. Specifically, we evaluated if the changes related to BOLD are triggered by interictal spikes in cortical structures that belong to physiological networks using a quantitative method. Finally, a group analysis of BOLD signal changes related to IED was conducted to search for a common network functionally altered TLE.

## Methods

### Participants

Patients affected by TLE were prospectively included in the study: 33 concluded the EEG–fMRI co-registration protocol (14 men; mean age 36 years, range 15–56 years).

The inclusion criteria were (a) patients ≥14 years old with an electro-clinical diagnosis of TLE; (b) patients entering a presurgical evaluation program; and (c) no contraindication to 3T MRI scanning.

This study was approved by the local Ethical Committee (N. 322/15) and written informed consent was obtained from all participants.

Table 1 summarizes the main electroclinical findings of the cohort of TLE patients. In 6 patients MRI was negative for epileptogenic lesions, whereas in the remaining 27 patients an epileptogenic lesion was observed. The most frequent radiological diagnosis was hippocampal sclerosis (*n* = 13), followed by low-grade tumors (*n* = 5) and focal cortical dysplasia (*n* = 4).

**Table 1:** Electro-clinical data of the patients with temporal lobe epilepsy included in the study.

	**TLE *n* = 33**
**Gender**, ***n***	
Female	19
Male	14
**Etiology**, ***n***	
Hippocampal sclerosis	13
Focal cortical dysplasia	4
Scar	1
Cavernoma/vascular	4
Low-grade tumors	5
MRI negative	6
Lesion Side^*^, ***n***	
Right	12
Left	14
Bilateral	1
MRI negative	6
Type of TLE^**^	
Mesial	26
Lateral	1
Age at study enrollment, years (range)	36 (15–56)
Age at epilepsy onset, years (range)	23.5 (4–49)
Duration of epilepsy, years (range)	12.6 (1–35)
Seizure frequency, *n*/month (range)	2.7 (0–20)
Seizures during wakefulness, *n* (%)	27 (82)
Seizure-Free periods^***^ *n* (%)	14 (42%)
Cluster seizures, *n* (%)	16 (48)
Focal aware seizures, *n* (%)	19 (58)
Focal impaired awareness seizures, *n* (%)	25 (76)
Focal to generalized seizures, *n* (%)	25 (76)
Status Epilepticus, *n* (%)	1 (3)
Falls during seizures, *n* (%)	11 (33)

* *Based on MRI findings. N.a., not applicable*.

** *Based on clinical and radiological findings. Sz/m, number of seizures per month. N pt, number of patients*.

*** *Period longer than 1 year without seizure recurrence*.

### Video-EEG–fMRI Protocol

Scalp EEG was recorded by means of a 32-channel MRI-compatible EEG recording system (Micromed, Mogliano Veneto, Italy). Electrodes were placed according to conventional 10–20 locations and the reference was FCz. ECG was recorded from two chest electrodes. Before scanning, 10 min of out-of-scanner EEG data were collected. Foam pads were used to help secure the EEG leads, minimize motion, and improve the comfort of the patient. Data were transmitted *via* an optic fiber cable from the high-input impedance amplifier (1,024/2,048 kHz sampling rate) to a computer located outside the scanner room. Patients were also constantly observed and recorded by a small camcorder positioned on the head coil inside the scanner pointing at the patient's face to obtain a split-screen video-EEG documentation during the fMRI recording (19, 20). Video data were used to monitor behavioral signs of sleep and movements.

Functional data were acquired using a Philips Achieva system at 3T and a gradient-echo echo-planar sequence from 30 axial contiguous slices (TR = 2,000 ms; in-plane matrix = 80 × 80; voxel size: 3 × 3 × 4) over one to three 8 min sessions per participant (240 volumes) with continuous video-EEG recording. The mean duration of the recording was 24 min (range 8–32 min), depending on the number of acquired sessions. In particular, one patient had four sessions recorded, 12 patients had three sessions, 18 patients had two sessions, and 2 patients had only one session. A high-resolution T1-weighted anatomical image was acquired for each participant to allow anatomical localization. The volume consisted of 170 sagittal slices (TR = 9.9 ms; TE = 4.6 ms; in-plane matrix = 256 × 256; voxel size = 1 × 1 × 1 mm).

### EEG Analysis

EEG acquisition was used to identify interictal (or ictal) epileptiform activity during the fMRI session. BrainQuick System Plus software (Micromed) was used for offline correction of the gradient artifacts and filtering of the EEG signal (21). In addition, the EEG data were exported in the.edf format and reviewed and analyzed by means of the BrainVision Analyzer 2.0 software (Brain Products, Munich, Germany). After removing the gradient and mean ballistocardiographic artifacts, according to the previously published methods (22), two experienced electroencephalographer reviewed the preprocessed EEG recordings (LM, AEV) to identify interictal epileptiform abnormalities based on both spatial distribution and topography. When recognized, IED were marked as intervals. We classified patients as unilateral (right or left) in case of only one spike focus without contralateral spreading; bilateral, in case both temporal lobes were active simultaneously (same timing of the left and right spikes). In the latter condition, left and right IED were considered together as a single event in the GLM analysis. In patients with independent bilateral temporal IED, both right and left IED were included in the design matrix, separately.

### Single-Subject Analysis

#### fMRI Analysis

The fMRI data analyses were performed using MATLAB version R2013a (The MathWorks Inc., Natick, MA, USA) and SPM12 (Wellcome Department of Imaging Neuroscience, London, UK). Preprocessing of functional volumes included slice timing correction, realignment to the first volume acquired, normalization to the MNI (Montreal Neurologic Institute) template implemented in SPM12 and smoothing with an 8 × 8 × 8 mm FWHM Gaussian kernel. The interictal (or ictal) epileptiform activity was implemented as a regressor of interest in the single patient-first level analysis. To do so, patients' IED were treated as a single event regardless of their topography or location. Their onset was exported in.mat file that describes the exact timing and duration (in seconds) of IED. The resulting timing files served as an onset for GLM, convolved with the standard hemodynamic response function (HRF) and its time and dispersion derivatives (TD, DD). Twenty-four motion parameters estimated during the realignment were included as nuisance regressors. The resulting fMRI maps (*F*-contrast) were estimated at the conventional statistical threshold of *p* < 0.05 at the voxel level (family-wise error (FWE)-corrected). In addition, in cases where the conventional FWE corrected statistical threshold did not show any results, the data were further explored with a less stringent statistical threshold of *p* < 0.001 (uncorrected for multiple comparisons). In the latter case, we applied a small volume correction (5 mm sphere) and we considered any BOLD activation/deactivation with a threshold of *p* < 0.05, FWE corrected.

This procedure reflects the standard procedure generally adopted in the previously published criteria (23–25). For each patient, specific contrasts were settled up to test the BOLD effect of IED with respect to the resting EEG background.

#### Identification of the Presumed EZ

The presumed EZ (pEZ) was defined based on the results of the presurgical work-up, which included video-EEG findings (interictal and ictal EEG activity and clinical ictal semiology), structural MRI scan, and interictal F-18 fluorodeoxyglucose FDG-PET when available. For each patient, this information was presented for discussion at a multidisciplinary team meeting, resulting in a consensus EZ localization. The EZ delineation and surgical decisions were blind with respect to the EEG–fMRI results. For all patients, the presumed EZ was localized in the temporal lobe.

#### Evaluation of Concordance

To evaluate the concordance between the map of BOLD changes related to IED and the presumed EZ, we applied previously published criteria (16, 26). Specifically, “Concordant” (C) refers to maps in which all the clusters (either activation or deactivation) are colocalized with the presumed EZ: within 2 cm of and in the same lobe as EZ; “Concordant Plus” (C+) is applied to fMRI maps with some clusters of significant IED-related BOLD changes colocalized with the pEZ and other significant BOLD clusters were located outside the pEZ (within or outside the temporal lobe). EEG–fMRI findings were defined “Discordant” (D) when all clusters of BOLD changes related to IED were remote from the pEZ and Null (N) where there was no cluster of significant BOLD change related to IED.

For patients with mesial TLE, the presumed EZ consisted of the cerebral structures classically removed by the standard anterior temporal lobectomy (27), that is, 6.0–6.5 cm of the anterior lateral non-dominant temporal lobe or 4.0–4.5 cm of the dominant temporal lobe. In these cases, medially, it included the amygdala and, at a minimum, the anterior 1.0–3.0 cm of the hippocampus (most commonly, 4.0 cm).

Note that the concordance between the map of BOLD changes related to IED and the presumed EZ was evaluated using non-normalized fMRI data.

#### Identification of ICN

To investigate if the cortical structures involved by the fMRI maps belonged to physiological networks (ICN), the *ICN_Atlas* toolbox was used to perform a quantitative analysis (28). This new methodology was designed to describe fMRI maps in a function-oriented way using a set of 15 metrics conceived to quantify the degree of “engagement” of ICNs for any given fMRI-derived statistical map of interest.

We used the BRAINMAP20, implemented in the ICN_Atlas toolbox, as an atlas base map. It includes 18 co-activation networks and 2 artifact components, and it is based on ICA decomposition (*d* = 20) of the BrainMap Project large-scale neuroimaging experiment meta-analysis data (29, 30).

For each subject, the ICN_Atlas output can reveal the involvement of one or more than one ICN with a different degree of “engagement.” The latter is reflected by a spatial involvement metric, the *I*_*j*_, which expresses the ratio between the number of activated voxels in the *i*th thresholded prototyped ICN map (ICN_*i*_) and the volume of ICN_*i*_ (29, 30).

For each patient, the spatial involvement results were visualized using rose plots (see **Figure 2** and Supplementary Figure 1). The axis limit is at the maximum of the subject-specific spatial involvement metric. There are tick marks at 25, 50, and 75% of the maximum.

We have calculated how many times each ICN was engaged in the group of patients with positive fMRI findings (total number = 19 patients). For each patient, the ICNs with a spatial involvement metric lower than the patient-specific 25% were not considered. The four most frequent ICNs were the motor (6/19), the visual (7/19), the auditory/motor speech (9/19), and the default mode networks (4/19).

To investigate the concordance between the most engaged ICNs (>patient-specific 25%) and ictal/postictal symptoms at the single-subject level, the corresponding ictal semiology (i.e., motor symptoms, such as tonic-clonic movements, visual auras, ictal, or postictal aphasia, impaired awareness) was examined. Ictal/postictal symptoms/signs refer both to clinical signs witnessed during video-EEG recording and to symptoms referred to by the patient in his/her recent clinical history (latest 3 months before the EEG–fMRI study).

Sensitivity, specificity, positive predictive value (PPV), and negative predictive value (NPV) of EEG–fMRI in detecting specific network alterations related to a corresponding symptom were evaluated.

Cases with an ICN involvement (i.e., motor network) and the presence of corresponding ictal/postictal semeiology (i.e., tonic-clonic seizures) were defined as true positive (TP), whereas true negative (TN) were the cases without ICNs engagement and absence of corresponding ictal/postictal semeiology (i.e., neither motor network nor motor symptoms); false positive (FP) were defined by the cases in which a specific ICN was involved but the patient did not present the corresponding clinical features (i.e., motor network involved, but absence of motor signs or symptoms), whereas false negative (FN) were defined by the cases without ICN involvement but with the presence of the corresponding symptoms (i.e., motor network not involved but patient referring recent tonico-clonic seizures).

The mathematical definition of sensitivity is given by the ratio between TP and the sum of TP and FN cases (= TP/TP + FN). Specificity corresponds to the ratio of TN and the sum of TN and FP (= TN/TN + FP). PPV represents the proportion of truly positive cases among the positive cases detected by the test (= TP/TP + FP), and NPV represents the proportion of truly negative cases among the negative cases detected by the test (= TN/TN + FN).

### Group Analysis

To ensure a homogeneous group with all patients having the seizure focus on the same side, functional volumes of right TLE patients were right–left flipped, so the ipsilateral hemisphere was on the left in all cases. This step was done using FSL (fslswapdim tool) before the preprocessing. Using the parameter estimates obtained by single-subject analyses, we performed a second-level (group) random-effect analyses on all patients with IEDs recorded during EEG–fMRI (*n* = 25). A full factorial design was used, with hemodynamic shapes (HRF, TD, and DD) as levels. Patients' age and gender were included in the model as covariates.

A double-statistical threshold (voxel-wise *p* < 0.001 and spatial extent of 15 voxels) was adopted to achieve a combined significance, corrected for multiple comparisons, of α < 0.05, as computed by 3dClustSim AFNI routine, using the “-acf” option (https://afni.nimh.nih.gov/pub/dist/doc/program_help /3dClustSim.html).

## Results

In 25 patients out of 33 (75%), epileptiform activity with morphology and scalp topography typical of patients' out-of-scan IEDs was identified. A mean of 16 IEDs/run per patient was recorded (range: 2–70). The number of sessions recorded did not influence the average number of IEDs/run: patients with 1 session had a mean of 15 IEDs/run, patients with 2 sessions had a mean of 20.6 IEDs/run, patients with 3 sessions had a mean of 9.7 IEDs/run and the patient with 4 sessions recorded had a mean of 13.2 IEDs/run (*p* = 0.1073, two-tailed *T*-test between session 2 and session 3). No seizures were recorded. In the remaining eight patients, no clear epileptiform discharges were found; therefore, the fMRI analysis could not be performed. In 19 patients spike-triggered BOLD signal changes were obtained, whereas six patients had a “Null” result (Supplementary Table 1).

### Presumed EZ and BOLD Concordance

**Figure 1:**
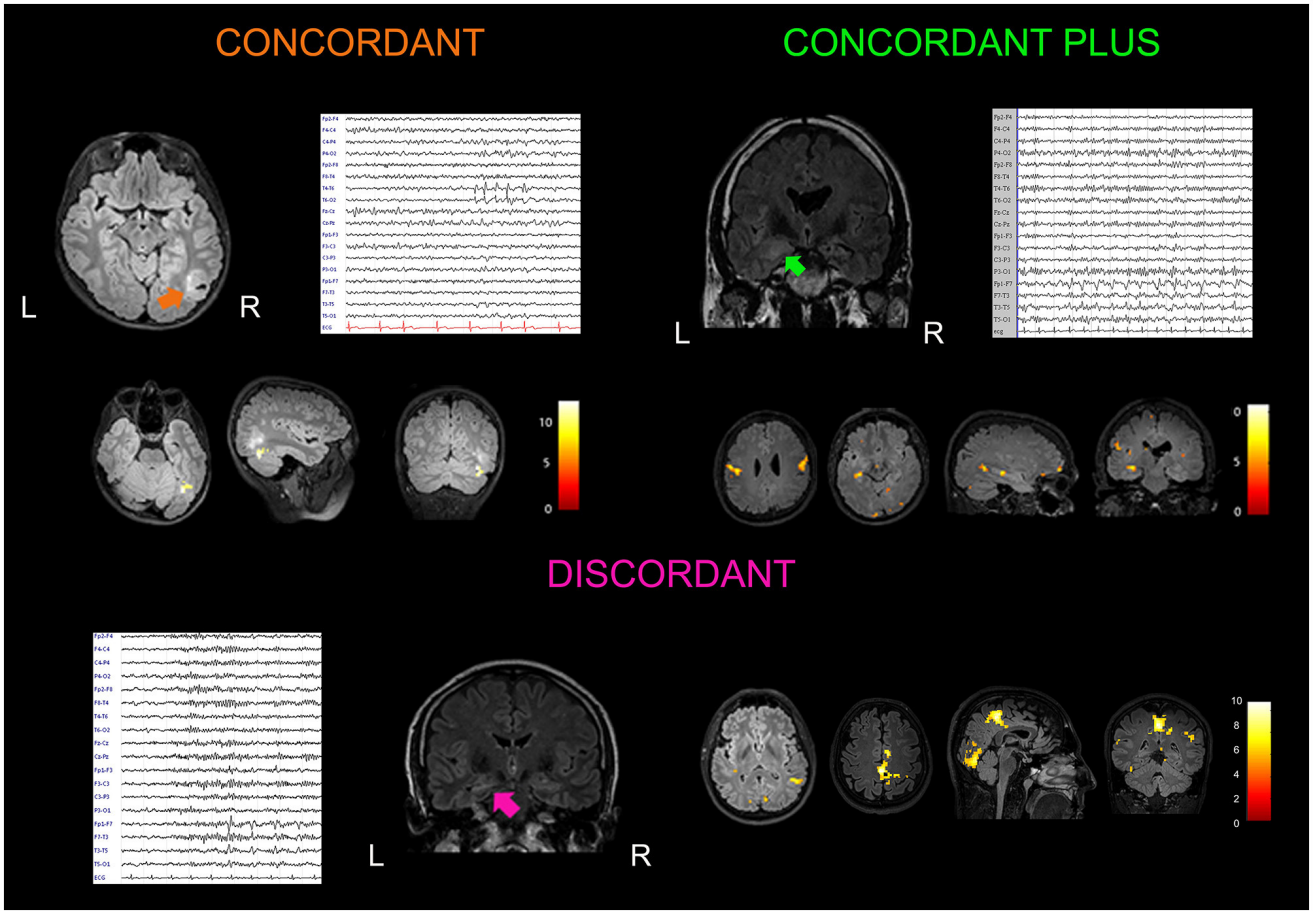
Examples of concordant, concordant plus and discordant EEG–fMRI findings. Concordant: A 15-year-old boy with a history of febrile seizures in early childhood presented with a focal to generalized seizure occurring during sleep. Brain MRI showed a lesion localized at the border of the temporal and occipital cortex on the right side (arrow). Low-amplitude spike and waves were observed in the right posterior temporal and occipital electrodes (T6, O2) on scalp EEG. He received surgery (lesionectomy) with a good outcome (Engel Class Ib at 24 months of follow up). Histological analysis documented a polymorphous low-grade neuroepithelieal tumor of the young (PLNTY). EEG–fMRI analysis guided by interictal T6 spike-and-waves showed a cluster of BOLD signal increment localized inside the tumor. Coronal, sagittal, and axial slices of the patient's structural MRI are displayed (*p* < 0.05), FWE corrected. Concordant plus: A 37-year-old woman presented with a 6-year history of focal seizures occurring on a weekly base. She referred to two types of seizures: one with an epigastric aura ascending from the lower abdomen to the neck, nausea, and brief impairment of awareness; second seizure type was a focal aware seizure with a visual aura (visual shrinking and oscillation) with dizziness sensation. Structural MRI showed two lesions suggestive for focal cortical dysplasia localized in the right anterior mesial temporal lobe, and in the posterior part of the middle temporal cortex. EEG confirmed two epileptic foci in the right temporal lobe, one anterior temporal (F8) and the other localized in the middle posterior temporal derivations (T4, T6). The patient refused surgery after seizures improvement with pharmacological treatment. EEG–fMRI showed BOLD changes in the right temporal lobe with a cluster in the vicinity of the posterior middle-temporal lesion, plus other clusters of increased BOLD changes localized at distant cerebral structures (ipsilateral and contralateral parietal, precentral, and occipital gyrus; *p* < 0.001, uncorrected for multiple comparisons). Discordant: A 40-year-old man with left hippocampal sclerosis presented frequent focal seizures characterized by epigastric aura, aphasia, and oral automatisms. Scalp EEG abnormalities were represented by infrequent spikes and waves visible in the anterior temporal derivations (F7, T3). Excellent seizure outcome (Engel Class Ia) was obtained after standard left anterior temporal lobectomy. EEG–fMRI showed widespread fMRI changes localized outside the left temporal lobe and involving cerebral structures belonging to the motor, visual, and DMNs (*p* < 0.001, uncorrected for multiple comparisons).

One patient, the only one with neo-cortical TLE, had a “Concordant” finding, 7 patients showed a “Concordant Plus” result and in the remaining 11 patients, fMRI maps were “Discordant” (58%). We considered patients with C and C+ results (*n* = 8, 42%) as a single group. Figure 1 shows three exemplificative cases. Differences in clinical, radiological, etiological, and surgery outcome data were evaluated between Concordant and Discordant patients. The only statistically significant difference (*p* = 0.02) regarded the antiepileptic treatment at the time of the neuroimaging study: a higher percentage of patients (75%, *n* = 6 out of 8 patients) with Concordant results were taking carbamazepine as an anti-seizure drug, compared to 18% of patients (*n* = 2 out of 11 patients) with Discordant results. At present, eight patients underwent surgery and all resulted in a good outcome (Engel class I): one had concordant and seven had discordant results (Supplementary Table 1).

### ICN Alterations Related to Spike

**Figure 2:**
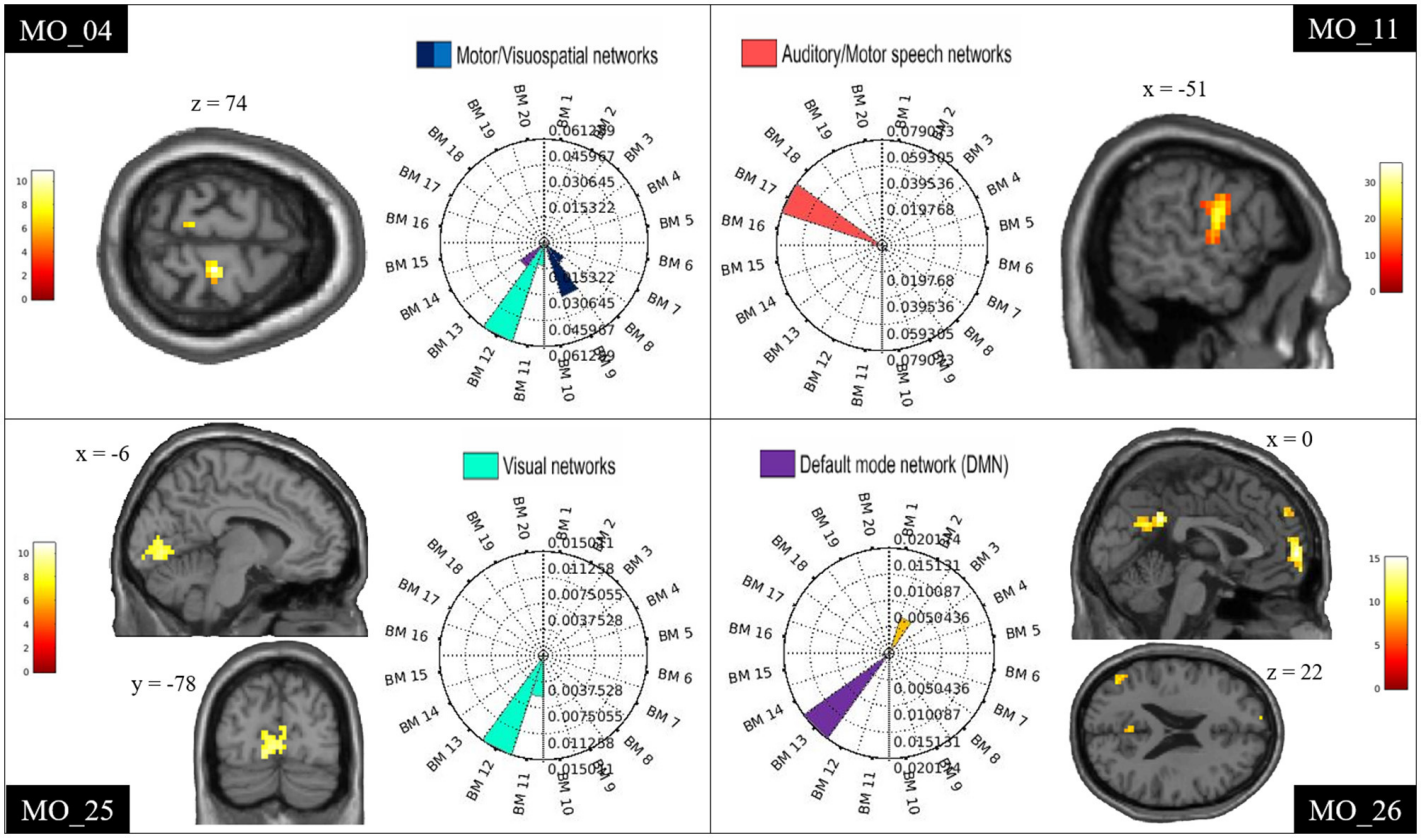
Examples of ICN alterations. Examples of single-subject results showing spike-triggered BOLD signal changes within the motor (MO_04), auditory/motor speech (MO_11), visual (MO_25), and DMN (MO_26). Clusters are shown in sagittal, coronal, and axial slices of an anatomical template as implemented in spm12. The rose plots represent the ICN_Atlas output: the spatial involvement metric expressing the ratio between the number of activated voxels and the volume of the ICNsi. BM refers to the co-activation networks of the BRAINMAP20 atlas base map.

In every patient with an fMRI map related to spike (*n* = 19), at least one ICN appeared involved (Table 2 and Figure 2). Supplementary Figure 1 reports the ICN involvement according to the *ICN_Atlas* for every other patient.

**Table 2:** Electro-clinical and radiological features of 19 patients with positive fMRI results.

**Patient**	**Age (years), sex**	**MRI findings**	**18-FDG PET hypo-metabolism**	**IEDs field**	**N**°**IEDs/run recorded**	**Surgery, outcome Engel class (follow up)**	**Resting state networks (ICN_atlas)**
1	37, F	R Focal cortical dysplasia	–	F8/T4	7	–	Visual
2	40, M	L Hippocampal sclerosis	–	F7/T3	23	ATL, Ia (24 months)	Visual; Motor; DMN
3	38, F	L Low grade tumor	–	F7/T3	7	Lesionectomy, Ib (12 months)	Visuospatial/motor; DMN; interoceptive
4	36, M	L Focal cortical dysplasia	–	F7/T3	7	–	Auditory/motor speech; visual
5	29, M	R Hippocampal sclerosis	–	F8/T4	22	ATL, Ia (15 months)	Visual; motor
6	22, F	L Hippocampal sclerosis	–	F7/T3/T5	8	–	Auditory/motor speech; motor
7	37, F	MRI negative	L temporo-parietal	T3/T5	9.5	–	DMN; auditory/motor speech
8	53, F	L Hippocampal sclerosis	–	F3/T3	33.5	–	L fronto-parietal; R fronto-parietal; cerebellum
9	36, M	L Low grade tumor	–	F7/T3/C3	27.5	ATL, Ia (20 months)	Visuospatial
10	56, M	MRI negative	–	F7/T3	10.3	–	Auditory/motor speech
11	29, F	R Hippocampal sclerosis	R temporal	F8/T4	1.7	ATL, Ia (26 months)	Auditory/motor speech; motor/ visuospatial
12	40, F	L Scar	–	F7/T3	5.5	–	DMN; emotion
13	43, F	R Low grade tumor	–	T4/T6	9	Lesionectomy, Ia (27 months)	Visual
14	42, M	MRI negative	–	F7/T3	13.3	–	Visual; motor; auditory/motor speech
15	46, M	R Hippocampal sclerosis	–	F8/T4	10	ATL, Ia (15 months)	L fronto-parietal
16	51, M	R Hippocampal sclerosis	R temporo-parietal	F8/T4/T6	10	–	Visuospatial
17	43, F	L Focal cortical dysplasia	–	F7/T3/T5	5	–	Auditory/motor speech
18	15, M	R Low grade tumor	–	T4/T6	70	Lesionectomy, Ib (23 months)	Visual
19	43, F	L Cavernoma	–	F7/T3	23	Lesionectomy, Ia (19 months)	Auditory/motor speech

*F, female; M, male; R, right; L, left; ATL, Anterior Temporal Lobectomy; FWE, Family-wise Error Corrected; unc, Uncorrected for Multiple Comparisons; DMN, Default Mode Network*.

The most frequent ICNs identified were, namely, motor, visual, auditory/motor speech, and DMN.

The specificity of EEG–fMRI analysis in detecting alteration of an ICN in patients with corresponding ictal/postictal symptoms was high for all four ICNs investigated (all above 65%), while the sensitivity of the test was high for the motor and the language networks, and lower for the other two networks (Table 3). In particular, for the motor network, sensitivity of 57% (n° of TP:4) was obtained, and specificity of 83% (n° of TN:10) was obtained; for the language network, sensitivity of 75% (n° of TP:6) and specificity of 73% (n° of TN:8) were obtained; for the visual network, sensitivity was of 50% (n° of TP:1) and specificity of 65% (n° of TN:11), respectively, and for the DMN, the sensitivity was of 25% (n° of TP:3), whereas the specificity resulted in 86% (n° of TN:6). The positive predictive value of the test was above 67% for all networks but the visual one. Negative predictive value was above 80% in all networks, with the exception of the DMN (Table 3).

**Table 3:** Concordance between Intrinsic Connectivity Networks (ICN) related to spike and seizure semeiology.

**Pt**	**Intrinsic connectivity networks (ICN_atlas)**				**Seizure semeiology**				**TLE side**
	**Visual**	**Motor**	**Auditory motor speech**	**Default mode network**	**Visual aura**	**Focal to general. sz**	**Aphasia**	**Impaired consciousness**	
1									R
2									L
3									L
4									L
5									R
6									L
7									L
8									L
9									L
10									L
11									R
12									L
13									R
14									L
15									R
16									R
17									L
18									R
19									L
	**Visual**	**Motor**	**Auditory motor speech**	**Default mode network**					
SENS	50%	57%	75%	25%	
SPEC	65%	83%	73%	86%	
PPV	14%	67%	67%	75%	
NPV	92%	77%	80%	40%	

*Pt, Number of Patient; TLE, temporal lobe epilepsy; meaning of colored cells: presence of ICN involvement or presence of clinical symptom in the patient; meaning of empty cells: absence of ICN or clinical symptom. SENS, Sensitivity; SPEC, Specificity; PPV, Positive Predictive Value; NPV, Negative Predictive Value*.

Moreover, we explored the existence of a correlation between the language network involvement and the side of TLE: the 88.9% (*n* = 8/9) of patients with language network involvement had TLE lateralized to the left.

### Group Results

**Figure 3:**
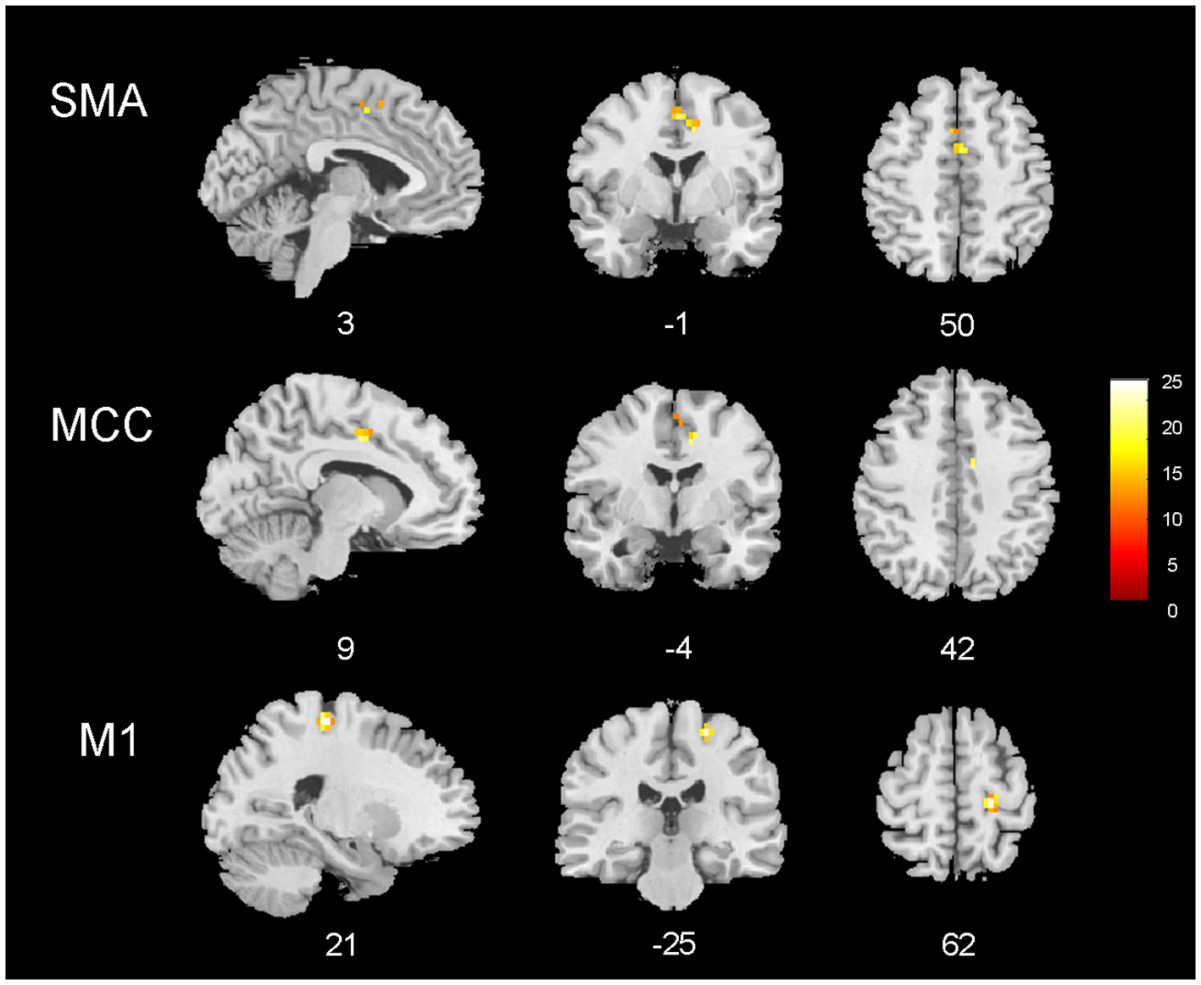
EEG–fMRI group analysis in 25 patients with temporal lobe epilepsy. Results of the group analysis (cluster size threshold *K* ≥ 15, corrected at α < 0.05). Clusters are shown in sagittal, coronal, and axial slices of an anatomical template found in the xjview toolbox (https://www.alivelearn.net/xjview). Color bar represents *t* values.

The group analysis including all patients (*n* = 25) revealed common BOLD changes related to IED at the right precentral gyrus, supplementary motor area (SMA), and middle cingulate gyrus. Regions showing BOLD signal changes time-locked to IEDs are summarized in Table 4 and Figure 3.

**Table 4:** Peak coordinates of group analysis.

**Cluster**	**Brain areas**	**BA**	**Voxel level**	**MNI coordinates**		
**K**			**Z**	**x**	**y**	**z**
15	Precentral gyrus	4	4.64	21	−25	62
23	Middle cingulate gyrus	24	4.24	9	−4	42
	Supplementary motor area	6	4.16	0	−1	50

*Cluster size threshold K ≥ 15, corrected at α < 0.05. BA, Brodmann Area*.

## Discussion

In this study, IED were recorded in 75% of the patients and in 58% a BOLD signal change was observed. These results are in line with the previous EEG–fMRI studies, confirming that a major limitation for the use of the technique in the presurgical setting in TLE patients is the low numbers of IEDs recording during EEG–fMRI (10), and in particular, for mesial TLE, which often generates infrequent scalp-detectable IEDs (31).

We have observed BOLD maps related to IED are concordant with the EZ only in one patient, affected by neocortical temporal lobe epilepsy (Concordant case, Figure 1), whereas in the remaining patients, we detected temporal lobe activations plus extratemporal areas in 7 patients, and only extratemporal activation in 11 patients. This finding questions the clinical utility of the presurgical interictal EEG–fMRI to localize the EZ in TLE patients. Compared to other studies, we observed less concordant results (3, 5, 7, 9). This might be related to the shorter duration (20–40 min) of our recordings compared to previous works performed by other groups [recordings lasting up to 84 min (7)], thus reducing the chance of detecting interictal abnormalities. These data suggest that in order to better evaluate patients with TLE, a longer EEG–fMRI session is required.

In agreement with a recent retrospective review investigating the clinical impact of EEG–fMRI in the presurgical setting (31), our results suggest that in patients with TLE short EEG–fMRI recordings are not useful for the surgical decision. When a clear epileptogenic lesion is detected on structural MRI, which matches the epileptogenic focus defined by the video-EEG data, EEG–fMRI might not add information for the surgical planning. This was the case for the 82% (*n* = 27) of the patients in our cohort, who had a positive MRI congruent with the electro-clinical data. In the remaining six patients with a negative MRI, in three patients no IED were recorded during EEG–fMRI, and in the other three cases, the fMRI response was represented by widespread changes of the BOLD signal.

In our cohort, BOLD signal changes occurred in the temporal lobe plus in extratemporal areas in 42% of cases, whereas only extra-temporal BOLD activations or deactivations were observed in the remaining 58% of patients. When comparing the two groups (concordant or concordant plus vs. discordant), no significant differences were found, and, in particular, no differences in seizure outcome were observed in patients who underwent surgery. This finding suggests that, at least in our series, multiple BOLD clusters related to IED involving regions outside the presumed EZ were not related to a worse surgery outcome.

To better understand the meaning of the BOLD response involving extratemporal cortical areas, we moved from a focus view to a network view, which is what a functional technique, such as EEG–fMRI, allows us to do, highlighting a patient's specific epileptic network. Indeed, advancement in functional neuroimaging leads to the observation that TLE is not only a disease of certain brain structures involved in seizures onset (“the focus”) but it also affects chronically the activity of brain networks that control basic functions, such as attention, emotion, and cognition (18, 32–34). Moreover, recent studies on patients with drug-resistant TLE enhanced functional differences in sub-group of patients through the use of diffusion tensor imaging or rs-fMRI, i.e., with or without focal to bilateral tonic-clonic seizures (FBTCS), especially in thalamo-cortical networks (35, 36).

Intrinsic connectivity networks can be altered in patients with TLE, as previously observed at group level (8, 18, 37–40). In this study, we were able to define IED-related ICNs alterations at a single-subject level, and to delineate and quantify the ICNs involvement. All patients had at least one ICN involved, and often more than one. According to the network classification of the BrainMap20 (28, 29), the most frequent BOLD changes involved the motor network, the visual network, the auditory/speech network, and the DMN. We looked for correlations between ICN alterations and clinical data, such as ictal or postictal semiological signs, and we found that in many patients with IED-related involvement of a distant ICN, ictal/postictal semiology was concordant with the function supported by that ICN. EEG–fMRI appeared to have high specificity (above 65%) for all 4 networks, while sensitivity was high for the language network (75%) and the motor network (57%) and lower for the visual network (50%), and the DMN (25%). Positive and negative predictive values were overall high, with the exception of the PPV low for the visual network, and NPV low for the DMN.

These findings confirm the role of EEG–fMRI in highlighting physiological networks alterations in patients with TLE, which might be possibly related to the clinical semeiology of the seizures. In other words, recurrent seizures with a specific ictal semeiology (i.e., aphasia) might interfere with the corresponding network (i.e., the language network) at the individual level, as revealed by spike-triggered EEG–fMRI.

A correct clinical testing of the seizure semeiology during video-EEG monitoring might play an important role: in fact, while motor and language deficits are easily recognized, the evaluation of consciousness is more complex. This might explain a reduction in sensitivity and NPV for the DMN.

Despite the relatively small number of patients enrolled, these observations represent a link between functional imaging data and the clinical seizures' phenotype. If confirmed in a larger group of patients, the research of ICN alterations through EEG–fMRI could represent complementary information to understand the fMRI maps, especially in complex cases. In particular, the finding of distant network involvement driven by interictal epileptic activity in patients with TLE, is of particular interest, underscoring the long-distance effects of interictal temporal lobe spikes.

Moreover, the correlation between the auditory/motor speech network involvement and side of TLE revealed that the great majority (n:8/9, 88.9%) had left-side TLE. This finding suggests that patients with the epileptogenic network localized in the left temporal lobe are more prone to have an effect on the language network, confirming data from previous studies of altered functional connectivity in the language network in left TLE patients (41).

With a second-level group analysis, we aimed to look for common spike-triggered functional alterations in patients with TLE. No BOLD changes were observed in the temporal structures, differently from the previous works (7, 8, 14). This finding is in line with the low frequency of temporal lobe fMRI changes observed at the single-subject level. Nevertheless, an involvement of extra-temporal structures belonging to the motor network (precentral gyrus and SMA) corroborates the alterations observed at the single-subject level in a significant proportion of cases. Since the motor network involvement is hemodynamically linked to the appearance of interictal temporal epileptic discharges on the scalp EEG, our findings suggest that IED might affect the neuronal activity of distant cerebral areas/networks subserving the sensory-motor functions. Interestingly, previous MRI morphometric studies have repeatedly reported a reduction of cortical thickness in the motor/premotor cortex in patients with TLE (42, 43). Thus, the present findings might represent the functional counterpart of these structural data although further data are needed to confirm this suggestion.

Finally, we observed an alteration of the BOLD signal in the middle cingulate gyrus, as previously described (8). In this work, the authors observed activation of mid-cingulate gyrus bilaterally in the group analysis of patients with TLE and also frontal lobe epilepsy (FLE), suggesting that the common involvement of the mid-cingulate gyrus in both groups might result from the rapid spread of epileptic activity from temporal and frontal areas. Moreover, the reduction in gray matter volume bilaterally in the cingulate gyri was observed in patients with TLE.

In conclusion, we suggest the use of EEG–fMRI co-registration for TLE patients, not only for localizing purposes but also to investigate ICN alterations. A larger number of patients are desirable to confirm our findings.

### Limitations of the Study

The present study presents methodological limitations that need to be underlined. First, fMRI data are driven by IED recorded from scalp EEG, not comprising the eventual intracranial epileptic activity that does not reach the cortical surface. This element might be considered a possible bias in the data interpretation.

As previously stated, TLE patients have infrequent IED which might be a limitation in the statistical power of the fMRI analysis: in fact, only a minority of patients presented BOLD cluster activations after FWE correction, whereas in the other patients the fMRI clusters were obtained only with *p* < 0.001 uncorrected. In the present study, the duration of EEG–fMRI registration was also shorter compared to other studies: this represents an important limitation, and also leads to the conclusion that in this group of patients longer EEG–fMRI registrations are needed especially for localizing purposes.

Moreover, the analysis performed at the group level included patients with heterogeneous features (i.e., different epilepsy etiologies, different disease durations, and different clinical semiologies) that might interfere with the final findings. Nevertheless, the results obtained in this group might represent the common epileptic network altered in TLE patients despite the heterogeneity.

Future studies including a larger number of patients might overcome these methodological limitations.

## Data Availability Statement

The raw data supporting the conclusions of this article will be made available by the authors, without undue reservation.

## Ethics Statement

The studies involving human participants were reviewed and approved by Comitato Etico Area Vasta Emilia Nord, Modena, Italy. Written informed consent to participate in this study was provided by the participants' legal guardian/next of kin.

## Author Contributions

LM was responsible for the data acquisition, analysis and interpretation of data, and drafting of the manuscript. DB and FT were responsible for the data acquisition, analysis and interpretation of data, revision of the manuscript, and final approval of the manuscript version to be published. GG and GP were responsible for the revision of the manuscript and final approval of the manuscript version to be published. AV was responsible for analysis and interpretation of data, revision of the manuscript, and final approval of the manuscript version to be published. SM was responsible for drafting/revision of the manuscript for content, including medical writing, study concept, and analysis and interpretation of data. All authors contributed to the article and approved the submitted version.

## Funding

This study was supported by a grant Dipartimenti di eccellenza 2018-2022, MIUR, Italy, to the Department of Biomedical, Metabolic and Neural Sciences, and by a grant Ricerca Finalizzata, project code NET-2013-02355313, Ministry of Health to the Azienda Ospedaliera-Universitaria di Modena Centro hub chirurgia epilessia (DGR 1172/18).

## Conflict of Interest

The authors declare that the research was conducted in the absence of any commercial or financial relationships that could be construed as a potential conflict of interest. The reviewer LC declared a shared consortium (ENIGMA-Epilepsy, ILAE imaging task force) with the authors AV and SM at time of review.

## Publisher's Note

All claims expressed in this article are solely those of the authors and do not necessarily represent those of their affiliated organizations, or those of the publisher, the editors and the reviewers. Any product that may be evaluated in this article, or claim that may be made by its manufacturer, is not guaranteed or endorsed by the publisher.

## References

[1] Krakov K Woermann FG Symms MR Allen PJ Lemieux L Barker GJ. EEG-triggered functional MRI of interictal epileptiform activity in patients with partial seizures. Brain. (1999) 122:1679–88. 10.1093/brain/122.9.1679 10468507

[2] Gotman J. Epileptic networks studied with EEG-fMRI. Epilepsia. (2008) 49:42–51. 10.1111/j.1528-1167.2008.01509.x 18304255 PMC3792078

[3] Kobayashi E Bagshaw AP Benar CG Aghakhani Y Andermann F Dubeau F. Temporal and extratemporal BOLD responses to temporal lobe interictal spikes. Epilepsia. (2006) 47:343–54. 10.1111/j.1528-1167.2006.00427.x 16499759

[4] Meletti S Vaudano AE Tassi L Caruana F Avanzini P. Intracranial time-frequency correlates of seizure-related negative BOLD response in the sensory-motor network. Clin Neurophysiol. (2015) 126:847–9. 10.1016/j.clinph.2014.07.030 25218363

[5] Pittau F Grouiller F Spinelli L Seeck M Michel C Vulliemoz. The role of functional neuroimaging in pre-surgical epilepsy evaluation. Front Neurol. (2014) 24:31. 10.3389/fneur.2014.00031 24715886 PMC3970017

[6] Salek-Haddadi A Diehl B Hamandi K Merschhemke M Liston A. Hemodynamic correlates of epileptiform discharges: an EEG-fMRI study of 63 patients with focal epilepsy. Brain Res. (2006) 1088:148–66. 10.1016/j.brainres.2006.02.098 16678803

[7] Kobayashi E Grova C Tyvaert L Dubeau F Gotman J. Structures involved at the time of temporal lobe spikes revealed by interindividual group analysis of EEG/fMRI data. Epilepsia. (2009) 50:2549–56. 10.1111/j.1528-1167.2009.02180.x 19552652 PMC3769286

[8] Fahoum F Lopes R Pittau F Dubeau F Gotman J. Widespread epileptic networks in focal epilepsies: EEG-fMRI study. Epilepsia. (2012) 53:1618–27. 10.1111/j.1528-1167.2012.03533.x 22691174 PMC4492710

[9] Pittau F Dubeau F Gotman J. Contribution of EEG/fMRI to the definition of the epileptic focus. Neurology. (2012) 78:1479–87. 10.1212/WNL.0b013e3182553bf7 22539574 PMC3345614

[10] Coan AC Chaudhary UJ Grouiller F Campos BM Perani S De Ciantis A. EEG-fMRI in the presurgical evaluation of temporal lobe epilepsy. J Neurol Neurosurg Psychiatry. (2016) 87:642–9. 10.1136/jnnp-2015-310401 26216941

[11] Vaudano AE Mirandola L Talami F Giovannini G Monti G Riguzzi P. fMRI-based effective connectivity in surgical remediable epilepsies: a pilot study. Brain Topogr. (2021) 34:632–50. 10.1007/s10548-021-00857-x 34152513

[12] Berg AT Berkovic SF Brodie MJ Buchhalter J Cross JH van Emde Boas W. Revised terminology and concepts for organization of seizures and epilepsies: report of the ILAE commission on classification and terminology, 2005-2009. Epilepsia. (2010) 51:676–85. 10.1111/j.1528-1167.2010.02522.x 20196795

[13] Avanzini G Manganotti P Meletti S Moshe SL Panzica F Wolf P. The system epilepsies: a pathophysiological hypothesis. Epilepsia. (2012) 53:771–8. 10.1111/j.1528-1167.2012.03462.x 22533642

[14] Laufs H Hamandi K Salek-Haddadi A Kleinschmidt AK Duncan JS Lemieux L. Temporal lobe interictal epileptic discharges affect cerebral activity in “default mode” brain regions. Hum Brain Mapp. (2007) 28:1023–32. 10.1002/hbm.20323 17133385 PMC2948427

[15] Grouiller F Thornton RC Groening K Spinelli L Duncan JS Schaller K. With or without spikes: localization of focal epileptic activity by simultaneous electroencephalography and functional magnetic resonance imaging. Brain. (2011) 134:2867–86. 10.1093/brain/awr156 21752790 PMC3656675

[16] Thornton R Laufs H Rodionov R Cannadathu S Carmichael DW Vulliemoz S. EEG correlated functional MRI and postoperative outcome in focal epilepsy. J Neurol Neurosurg Psychiatry. (2010) 81:922–7. 10.1136/jnnp.2009.196253 20547617

[17] Raichle ME MacLeod AM Snyder AZ Powers WJ Gusnard DA Shulman GL. A default mode of brain function. Proc Natl Acad Sci USA. (2001) 98:676–82. 10.1073/pnas.98.2.676 11209064 PMC14647

[18] Cataldi M Avoli M de Villers-Sidani E. Resting state networks in temporal lobe epilepsy. Epilepsia. (2013) 54:2048–59. 10.1111/epi.12400 24117098 PMC4880458

[19] Chaudhary UJ Rodionov R Carmichael DW Thornton RC Duncan JS Lemieux L. Improving the sensitivity of EEG-fMRI studies of epileptic activity by modelling eye blinks, swallowing and other video-EEG detected physiological confounds. Neuroimage. (2012) 61:1383–93. 10.1016/j.neuroimage.2012.03.028 22450296

[20] Ruggieri A Vaudano AE Benuzzi F Serafini M Gessaroli G Farinelli V. Mapping (and modeling) physiological movements during EEG-fMRI recordings: the added value of the video acquired simultaneously. J Neurosci Methods. (2015) 239:223–37. 10.1016/j.jneumeth.2014.10.005 25455344

[21] Allen PJ Josephs O Turner R. A method for removing imaging artifact from continuous EEG recorded during functional MRI. Neuroimage. (2000) 12:230–9. 10.1006/nimg.2000.0599 10913328

[22] Vaudano AE Ruggieri A Vignoli A Avanzini P Benuzzi F Gessaroli G. Epilepsy-related brain networks in ring chromosome 20 syndrome: an EEG-fMRI study. Epilepsia. (2014) 55:403–13. 10.1111/epi.12539 24483620

[23] Siniatchkin M Groening K Moehring J Moeller F Boor R Brodbeck V. Neuronal networks in children with continuous spikes and waves during slow sleep. Brain. (2010) 133:2798–813. 10.1093/brain/awq183 20688812

[24] Moeller F Stephani U Siniatchkin M. Simultaneous EEG and fMRI recordings (EEG-fMRI) in children with epilepsy. Epilepsia. (2013) 54:971–82. 10.1111/epi.12197 23647021

[25] Meletti S Ruggieri A Avanzini P Caramaschi E Filippini M Bergonzini P. Extrastriate visual cortex in idiopathic occipital epilepsies: The contribution of retinotopic areas to spike generation. Epilepsia. (2016) 57:896–906. 10.1111/epi.13385 27093945

[26] Markoula S Chaudhary UJ Perani S De Ciantis A Yadee T Duncan JS. The impact of mapping interictal discharges using EEG-fMRI on the epilepsy presurgical clinical decision making process: A prospective study. Seizure. (2018) 61:30–7. 10.1016/j.seizure.2018.07.016 30059825

[27] Wiebe S Blume W Girvin JP Eliasziv M. A randomized, controlled trial of surgery for temporal lobe epilepsy. NEJM. (2001) 345:311–8. 10.1056/NEJM200108023450501 11484687

[28] Kozák LR van Graan LA Chaudhary UJ Szabó ÁG Lemieux L. ICN_Atlas: Automated description and quantification of functional MRI activation patterns in the framework of intrinsic connectivity networks. Neuroimage. (2017) 163:319–41. 10.1016/j.neuroimage.2017.09.014 28899742 PMC5725313

[29] Laird AR Lancaster JL Fox PT. BrainMap: the social evolution of a human brain mapping database. Neuroinformatics. (2005) 3:65–78. 10.1385/NI:3:1:065 15897617

[30] Laird AR Fox PM Eickhoff SB Turner JA Ray KL McKay DR. Behavioral interpretations of intrinsic connectivity networks. J Cogn Neurosci. (2011) 23:4022–37. 10.1162/jocn_a_00077 21671731 PMC3690655

[31] Kowalczyk MA Omidvarnia A Abbott DF Tailby C Vaughan DN Jackson GD. Clinical benefit of presurgical EEG-fMRI in difficult-to-localize focal epilepsy: A single-institution retrospective review. Epilepsia. (2020) 61:49–60. 10.1111/epi.16399 31792958

[32] Trimmel K Vos SB Caciagli L Xiao F van Graan LA Winston GP. Decoupling of functional and structural language networks in temporal lobe epilepsy. Epilepsia. (2021) 62:2941–54. 10.1111/epi.17098 34642939 PMC8776336

[33] Fadaie F Lee HM Caldairou B Gill RS Sziklas V Crane J. Atypical functional connectome hierarchy impacts cognition in temporal lobe epilepsy. Epilepsia. (2021) 62:2589–603. 10.1111/epi.17032 34490890

[34] Li X Jiang Y Li W Qin Y Li Z Chen Y. Disrupted functional connectivity in white matter resting-state networks in unilateral temporal lobe epilepsy. Brain Imaging Behav. (2021). 10.1007/s11682-021-00506-8 34478055

[35] Chen C Li H Ding F Yang L Huang P Wang S. Alterations in the hippocampal-thalamic pathway underlying secondarily generalized tonic-clonic seizures in mesial temporal lobe epilepsy: A diffusion tensor imaging study. Epilepsia. (2019) 60:121–30. 10.1111/epi.14614 30478929

[36] He X Chaitanya G Asma B Caciagli L Basset DS Tracy JI. Disrupted basal ganglia-thalamocortical loops in focal to bilateral tonico-clonic seizures. Brain. (2020) 143:175–90. 10.1093/brain/awz361 31860076 PMC7954388

[37] Zhang Z Lu G Zhong Y Tan Q Liao W Wang Z. Altered spontaneous neuronal activity of the default-mode network in mesial temporal lobe epilepsy. Brain Res. (2010) 1323:152–60. 10.1016/j.brainres.2010.01.042 20132802

[38] Liao W Zhang Z Pan Z Mantini D Ding J Duan X. Default mode network abnormalities in mesial temporal lobe epilepsy: a study combining fMRI and DTI. Hum Brain Mapp. (2011) 32:883–95. 10.1002/hbm.21076 20533558 PMC6870458

[39] Pittau F Grova C Moeller F Dubeau F Gotman J. Patterns of altered functional connectivity in mesial temporal lobe epilepsy. Epilepsia. (2012) 53:1013–23. 10.1111/j.1528-1167.2012.03464.x 22578020 PMC3767602

[40] Tong X An D Xiao F Lei D Niu R Li W. Real-time effects of interictal spikes on hippocampus and amygdala functional connectivity in unilateral temporal lobe epilepsy: An EEG-fMRI study. Epilepsia. (2019) 60:246–54. 10.1111/epi.14646 30653664

[41] Trimmel K van Graan AL Caciagli L Haag A Koepp MJ Thompson PJ. Left temporal lobe language network connectivity in temporal lobe epilepsy. Brain. (2018) 141:2406–18. 10.1093/brain/awy164 29939211

[42] Whelan CD Altmann A Botía JA Jahanshad N Hibar DP Absil J. Structural brain abnormalities in the common epilepsies assessed in a worldwide ENIGMA study. Brain. (2018) 141:391–408. 10.1093/brain/awx341 29365066 PMC5837616

[43] Bernasconi N Duchesne S Janke A Lerch J Collins DL Bernasconi A. Whole-brain voxel-based statistical analysis of gray matter and white matter in temporal lobe epilepsy. Neuroimage. (2004) 23:717–23. 10.1016/j.neuroimage.2004.06.015 15488421

