# Supplementary material for: Temporal Lobe Spikes Affect Distant Intrinsic Connectivity Networks
Source: Front Neurol. 2021 Dec 17;12:746468. doi: 10.3389/fneur.2021.746468 (PMC8718871; doi:10.3389/fneur.2021.746468)
Supplement: Supplementary file 1 [file Table_1.DOCX]

**Table 1 s: Differences in clinical and radiological features between the groups of patients with Concordant fMRI results and Discordant fMRI results.**

|  | **Concordant**  **(n=8)** | **Discordant**  **(n=11)** | **p-value** |
| --- | --- | --- | --- |
| ***Age***  Mean ± SD | 35±11.7 | 41±8.2 | 0.17 |
| ***Gender, n (%)***  Male  Female | 3 (37.5)  5 (62.5) | 6 (54.5)  5 (45.5) | 0.65 |
| ***Clinical variables, n (%)***  History of febrile seizures  Age of epilepsy onset  Duration of epilepsy  Frequent seizures^*^  Cluster  Focal aware seizures  Focal seizures with awareness impairment  Focal to generalized seizures  Carbamazepine^**^ | 1 (12.5)  18.4±10.7  15.4±9.8  2 (25)  5 (62.5)  3 (37.5)  8 (100)  6 (75)  6 (75) | 3 (27.3)  26.1±15.2  15.4±11.3  4 (36.4)  5 (45.5)  9 (81.8)  8 (72.7)  8 (72.7)  2 (18.2) | 0.60  0.23  0.1  1.0  0.64  0.07  0.22  1  **0.02** |
| ***Radiological variables, n (%)***  *Side*  Right  Left  Bilateral  Negative  *Diagnosis*  Hippocampal sclerosis  Focal cortical dysplasia  Tumor  Cavernoma  Negative | 2 (25)  4 (50)  0  2 (25)  2 (25)  2 (25)  2 (25)  0  2 (25) | 4 (36.4)  5 (45.5)  1 (9.1)  1 (9.1)  5 (45.5)  2 (18.2)  2 (18.5)  1(9.1)  1(9.1) | 1  1  1  0.6  0.6  1  1  1  0.5 |
| ***Surgical outcome, n^***^***  Engel Class Ia  Engel Class Ib | 0  1 | 7  0 | 0.125  0.125 |

*: Frequent seizures > 3 seizures/month, **: number of patients assuming carbamazepine at the time of MRI study, ***: A total of 1 patient received surgery in the concordant group, and 7 patients in the discordant group.

**Figure 1 s:**  **Intrinsic Connectivity Network (ICN) involved in patients with a spike-related fMRI map. The rose plots of MO_04-11-25-26 patients are shown in Figure 2. The networks refer to the BRAINMAP20 atlas, implemented in the ICN_Atlas toolbox**.^29-30^

**
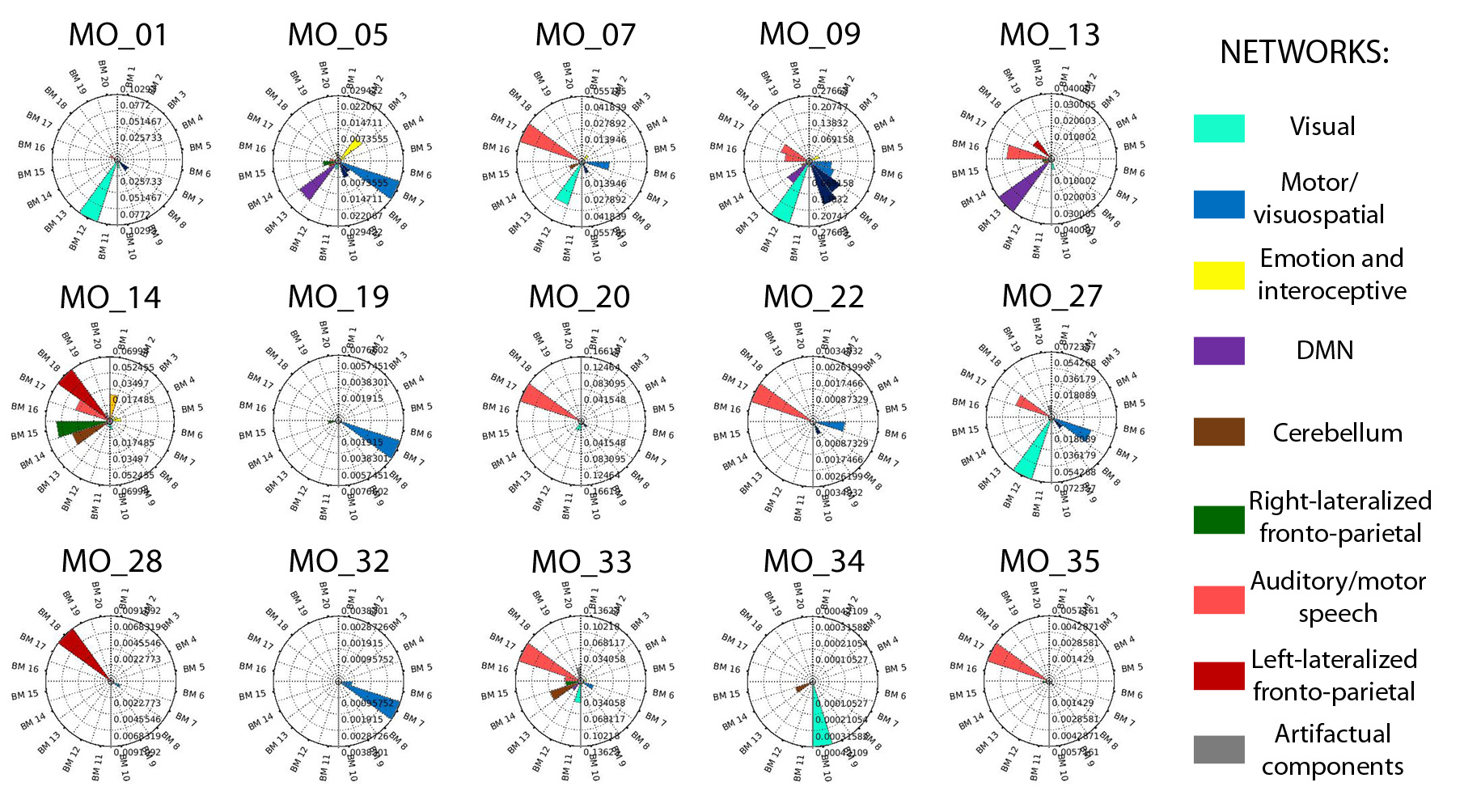
**

**Table 2 s: Clusters of spike- triggered fMRI changes at single subject level.**

| Pt | Regions | BA | Lateralization | Activation(+)/  Deactivation(-) | NI coordinates | | | Z score |
| --- | --- | --- | --- | --- | --- | --- | --- | --- |
|  |  |  |  |  | x | y | z |  |
| 01  *p<0.001 unc.* | Occipital Lobe, Cuneus | 30 | R | + | 3 | -67 | 6 | 5 |
|  | Cerebellum, Declive | / |  |  | 18 | -70 | -14 | 4,7 |
|  | Occipital Lobe, Middle Occipital Gyrus | 19 | R | + | 51 | -61 | -6 | 4,5 |
|  | Temporal Lobe, Inferior Temporal Gyrus | 37 |  |  | 51 | -52 | -6 | 4,4 |
|  | Parietal Lobe, Postcentral Gyrus | 3 | L | + | -33 | -28 | 70 | 4,5 |
|  |  | 2 |  |  | -51 | -25 | 42 | 4,3 |
|  |  | 1 |  |  | -48 | -22 | 58 | 4,1 |
|  | Parietal Lobe, Postcentral Gyrus | 3 | R | + | 51 | -25 | 62 | 4 |
|  |  | 2 |  |  | 60 | -22 | 50 | 3,9 |
|  | Parietal Lobe, Postcentral Gyrus | 4 | L | + | -57 | -13 | 34 | 3,4 |
| 04  *p<0.001 unc* | Frontal Lobe, Paracentral Gyrus | 5 | L | + | 15 | -28 | 74 | 4,8 |
|  | Frontal Lobe, Medial Frontal Gyrus | 6 |  |  | -3 | -19 | 54 | 4,5 |
|  | Temporal Lobe,Superior Temporal Gyrus | 13 | R | - | 51 | -40 | 18 | 4,5 |
|  | Parietal Lobe, Supramarginal Gyrus | 40 |  |  | 48 | -40 | 30 | 7,8 |
|  | Cerebellum, Declive | / | L | + | 0 | -76 | -6 | 4,3 |
|  | Ocipital Lobe, Cuneus | 23 | R |  | 6 | -73 | 10 | 4 |
|  | Occipital Lobe, Lingual Gyrus | 17 | L |  | -12 | -94 | -14 | 4 |
| 05  *p<0.001 unc* | Caudate | / | L | + | -18 | -19 | 22 | 5,2 |
|  | Thalamus |  | R |  | 6 | -10 | 22 | 4 |
|  | Frontal Lobe, Inferior Frontal Gyrus | 47 | L | - | -51 | 26 | -18 | 4,1 |
|  | Temporal Lobe,Superior Temporal Lobe | 38 |  |  | -57 | 17 | -30 | 4 |
|  | Parietal Lobe, Inferior Parietal Lobule | 40 | R | + | 45 | -43 | 58 | 4,6 |
|  | Parietal Lobe, Postcentral Gyrus | 5 |  |  | 42 | -43 | 58 | 4,4 |
|  | Parietal Lobe, Precuneus | 19 |  |  | 27 | -79 | 38 | 4,3 |
|  | Parietal Lobe, Inferior Parietal Lobule | 40 | L | + | -57 | -58 | 42 | 4,4 |
|  | Parietal Lobe, Angular Gyrus | 39 |  |  | -51 | -67 | 34 | 4,2 |
|  | Occipital Lobe, Superior Occipital Gyrus | 19 |  |  | -39 | -73 | 22 | 4,1 |
|  | Parietal Lobe, Postcentral Gyrus | 2 | L | + | -36 | -34 | 62 | 4,4 |
|  | Frontal Lobe, Precentral Gyrus | 4 |  |  | -36 | -25 | 62 |  |
| 07  *p<0.001 unc* | Posterior Cingulate | 30 | L | + | -18 | -58 | 6 | 5,4 |
|  | Occipital Lobe, Cuneus | 19 |  |  | -15 | -79 | 30 | 5 |
|  |  | 23 |  |  | -12 | -70 | 10 | 4,5 |
|  | Frontal Lobe, Precentral Gyrus | 4 | R | + | 57 | -7 | 26 | 5,1 |
|  | Parietal Lobe, Postcentral Gyrus | 3 |  |  | 57 | -16 | 22 | 4,1 |
|  | Cerebellum, Posterior Lobe | / | R | - | 30 | -82 | -34 | 5,1 |
|  | Frontal Lobe, Superior Frontal Gyrus | 6 | L | + | -3 | 5 | 62 | 5 |
|  | Parietal Lobe, Postcentral Gyrus | 7 | R | + | 3 | -52 | 70 | 4,7 |
|  |  | 5 | L |  | 0 | -43 | 70 | 3,9 |
|  | Ippocampi | / | L | - | -30 | -25 | -10 | 4,7 |
|  | Temporal Lobe, Sub-Gyral |  |  |  | -36 | -31 | -2 | 4,1 |
|  | Parietal Lobe, Postcentral Gyrus | 43 | L | + | -54 | -7 | 22 | 4,6 |
|  | Frontal Lobe, Precentral Gyrus | 6 |  |  | -54 | -4 | 30 | 4,5 |
|  | Parietal Lobe, Postcentral Gyrus | 40 |  |  | -54 | -22 | 22 | 4,5 |
|  | Thalamus | / | R | - | 12 | -10 | 18 | 4,5 |
|  | Caudate Body |  |  |  | 15 | -1 | 22 | 4,4 |
|  | Parahippocampal Gyrus | 30 | L | - | -15 | -37 | 6 | 4,5 |
|  | Caudate | / |  |  | -12 | -22 | 22 | 4,1 |
| 09  *p<0.001 unc* | Occipital Lobe, Cuneus | 19 | L | + | -6 | -79 | 34 | 5,2 |
|  | Parietal Lobe, Postcentral Gyrus | 3 | R |  | 30 | -31 | 70 | 5 |
|  | Frontal Lobe, Superior Frontal Gyrus | 8 | R | + | 27 | 35 | 50 | 4,3 |
|  | Temporal Lobe, Middle Temporal Lobe | 21 | L | + | -57 | -58 | 6 | 4,3 |
|  |  | 37 |  |  | -57 | -64 | -2 | 3,9 |
|  | Thalamus | / | L | + | 0 | -19 | 10 | 4,1 |
|  | Cerebellum, Posterior Lobe | / | R | + | 33 | -76 | -30 | 4,1 |
|  | Frontal Lobe, Middle Frontal Gyrus | 10 | R | + | 30 | 44 | 26 | 4 |
|  |  | 9 |  |  | 27 | 56 | 30 | 3,9 |
|  | Putamen | / | L | + | -24 | 8 | -2 | 3,9 |
|  | Caudate Body |  |  |  | -15 | 11 | 6 | 3,6 |
|  | Frontal Lobe, Inferior Frontal Gyrus | 47 | L | + | -54 | 23 | -2 | 3,8 |
|  |  | 45 |  |  | -51 | 35 | 2 | 3,6 |
|  | Temporal Lobe, Superior Temporal Gyrus | 38 |  |  | -57 | 14 | -10 | 3,6 |
| 11  *p<0.05 FWE* | Frontal Lobe, Precentral Gyrus | 6 | R | + | 54 | -7 | 26 | Inf |
|  | Insula | 13 |  |  | 36 | -13 | 14 | 5,2 |
|  | Frontal Lobe, Precentral Gyrus | 6 | L | + | -51 | -7 | 22 | Inf |
|  |  | 4 |  |  | -39 | -19 | 38 | 7,8 |
|  | Temporal Lobe, Superior Temporal Gyrus | 22 |  |  | -54 | -10 | 6 | 6,6 |
|  | Insula | 13 | L | + | -39 | -10 | 10 | 5,8 |
|  | Parietal Lobe, Postcentral Gyrus | 4 | R | + | 18 | -34 | 58 | 5,2 |
| 13  *p<0.001 unc* | Temporal Lobe, Middle Temporal Lobe | 21 | R | - | 63 | -40 | -6 | 6 |
|  | Parietal Lobe, Supramarginal Gyrus | 40 | L | - | -54 | -49 | 34 | 5 |
|  | Temporal Lobe, Superior Temporal Gyrus | 13 |  |  | -45 | -46 | 22 | 4,8 |
|  | Temporal Lobe, Middle Temporal Lobe | 21 | L | - | -63 | -52 | -6 | 5 |
|  | Cerebellum, Declive | / | R | - | 51 | -70 | -18 | 4,4 |
| 17  *p<0.001 unc* | Temporal Lobe, Inferior Temporal Gyrus | 20 | L | + | -45 | -13 | -34 | 6,3 |
|  | Frontal Lobe, Middle Frontal Gyrus | 10 | L |  | -39 | 56 | -6 | 5,1 |
|  | Frontal Lobe, Middle Frontal Gyrus | 10 | R | - | 39 | 56 | -2 | 5,4 |
|  |  | 11 |  |  | 42 | 47 | -10 | 3,6 |
|  | Frontal Lobe, Precentral Gyrus | 4 | L | + | -63 | -7 | 22 | 5,2 |
|  | Frontal Lobe, Inferior Frontal Gyrus | 9 |  |  | -48 | 5 | 30 | 4,3 |
|  |  | 44 |  |  | -60 | 8 | 18 | 3,7 |
|  | Cerebellum, Posterior Lobe | / | L | + | -45 | -70 | -38 | 5,2 |
|  | Frontal Lobe, Middle Frontal Gyrus | 9 | R | - | 48 | 17 | 30 | 5 |
|  |  | 46 |  |  | 51 | 29 | 18 | 4,2 |
|  |  | 6 |  |  | 45 | 11 | 46 | 4,2 |
|  | Frontal Lobe, Precentral Gyrus | 6 | R | + | 54 | -7 | 30 | 5 |
|  | Cerebellum, Posterior Lobe | / | R | + | 21 | -31 | -42 | 4,6 |
| 19  *p<0.001 unc* | Parietal Lobe, SuperiorParietal Lobule | 7 | R | + | 30 | -73 | 46 | 4,3 |
| 20  *p<0.05 FWE* | Frontal Lobe, Precentral Gyrus | 6 | L | + | -51 | -7 | 34 | Inf |
|  |  | 4 |  |  | -45 | -16 | 38 | 7,6 |
|  | Parietal Lobe, Postcentral Gyrus | 3 |  |  | -39 | -22 | 58 | 7,4 |
|  | Parietal Lobe, Postcentral Gyrus | 3 | R | + | 54 | -10 | 42 | Inf |
|  | Frontal Lobe, Precentral Gyrus | 6 |  |  | 51 | -4 | 26 | Inf |
|  | Occipital Lobe, Cuneus | 19 | R | + | 12 | -91 | 22 | 6,9 |
|  |  |  | L |  | 0 | -76 | 34 | 6,5 |
|  |  | 18 | R |  | 3 | -76 | -10 | 6,3 |
|  | Occipital Lobe, Inferior Occipital Gyrus | 18 | L | + | -39 | -85 | -10 | 6,9 |
|  | Occipital Lobe, Middle Occipital Gyrus | 19 |  |  | -51 | -73 | -6 | 6..85 |
|  | Occipital Lobe, Inferior Occipital Gyrus | 18 | R | + | 42 | -82 | -14 | 6,8 |
|  | Occipital Lobe, Middle Occipital Gyrus |  |  |  | 36 | -88 | -2 | 6,6 |
|  | Occipital Lobe, Lingual Gyrus | 19 | R | + | 18 | -43 | -2 | 6,4 |
|  | Cerebellum, Culmen | / |  |  | 15 | -52 | -6 | 5,2 |
|  | Parahippocampal Gyrus | 30 |  |  | 9 | -37 | 6 | 5 |
|  | Putamen | / | L | + | -24 | 5 | -2 | 6,2 |
|  | Cerebellum, Declive | / | R | + | 30 | -73 | -18 | 6 |
|  | Frontal Lobe, Medial Frontal Gyrus | 6 | R | + | 6 | -1 | 58 | 6 |
|  | Occipital Lobe, Inferior Occipital Gyrus | 37 | R | + | 54 | -67 | 2 | 5,8 |
|  | Temporal Lobe, Fusiform Gyrus |  |  |  | 51 | -61 | -10 | 4,9 |
|  | Cerebellum, Declive | / | L | + | -15 | -67 | -14 | 5,7 |
|  | Claustrum | / | R | + | 36 | -7 | 10 | 5,7 |
|  | Frontal Lobe, Middle Frontal Gyrus | 10 | R | + | 30 | 50 | 18 | 5,6 |
|  | Temporal Lobe, Superior Temporal Gyrus | / | R | + | 54 | 2 | 2 | 5,6 |
|  | Isula | 13 | L | + | -39 | -1 | 10 | 5,5 |
|  | Posterior Cingulate | 30 | R | + | 15 | -61 | 10 | 5,4 |
|  | Cingulate Gyrus | 24 | R | + | 9 | -1 | 46 | 5,4 |
|  | Putamen | / | R | + | 30 | -16 | 6 | 5,3 |
| 22  *p<0.001 unc* | Frontal Lobe, Precentral Gyrus | / | R | + | 3 | -19 | 74 | 3,8 |
| 25  *p<0.001 unc* | Frontal Lobe, Medial Frontal Gyrus | 10 | L | - | 0 | 62 | 2 | 4,8 |
|  | Frontal Lobe, Superior Frontal Gyrus |  |  |  | -3 | 68 | -10 | 4,1 |
|  | Frontal Lobe, Superior Frontal Gyrus | 9 | L | - | -18 | 53 | 34 | 4,8 |
|  |  | 8 |  |  | -18 | 47 | 42 | 4 |
|  | Precuneus | 31 | L | - | 0 | -46 | 30 | 4,6 |
|  | Cingulate Gyrus |  |  |  | 0 | -58 | 26 | 4,1 |
| 26  *p<0.05 FWE* | Occipital Lobe, Lingual Gyrus | 17 | L | - | -9 | -94 | -6 | 5,9 |
|  |  | 18 |  |  | -9 | -82 | -6 | 5,6 |
|  | Occipital Lobe, Cuneus |  | R |  | 6 | -85 | 14 | 5,4 |
|  | Parahippocampal Gyrus | 30 | L | + | -15 | -40 | 6 | 5,2 |
|  | Occipital Lobe, Lingual Gyrus | 19 | L | - | -21 | -64 | -2 | 4,9 |
|  | Cerebellum, Culmen | / |  |  | -12 | -67 | -10 | 4,9 |
| 27  *p<0.001 unc* | Frontal Lobe, Superior Frontal Gyrus | 8 | R | + | 24 | 35 | 54 | 5,4 |
|  | Precuneus | 7 | R | + | 6 | -58 | 54 | 5,1 |
|  | Parietal Lobe, Postcentral Gyrus |  |  |  | 18 | -55 | 70 | 3,3 |
|  | Parietal Lobe, Superior Parietal Lobule |  |  |  | 15 | -52 | 58 | 3,3 |
|  | Frontal Lobe, Precentral Gyrus | 6 | L | + | -45 | -7 | 30 | 5 |
|  | Parietal Lobe, Postcentral Gyrus | 3 |  |  | -51 | -10 | 50 | 3,9 |
|  | Parietal Lobe, Superior Parietal Lobule | 7 | R | + | 30 | -76 | 46 | 4,8 |
|  | Precuneus | 19 |  |  | 24 | -82 | 46 | 4,6 |
|  | Occipital Lobe, Cuneus | 18 | L |  | 0 | -79 | 22 | 4,5 |
|  | Parietal Lobe, Superior Parietal Lobule | 7 | L | + | -36 | -49 | 62 | 4,8 |
|  | Frontal Lobe, Precentral Gyrus | 6 | R | + | 57 | -1 | 18 | 4,3 |
|  |  | 44 |  |  | 60 | 8 | 6 | 3,7 |
|  | Parietal Lobe, Inferior Parietal Lobule | 40 | R | + | 42 | -37 | 54 | 4,1 |
|  | Parietal Lobe, Postcentral Gyrus | 2 |  |  | 48 | -28 | 58 | 4 |
|  | Occipital Lobe, Middle Occipital Gyrus | 19 | L | + | -57 | -67 | -6 | 4,1 |
|  | Occipital Lobe, Inferior Occipital Gyrus | 19 |  |  | -48 | -79 | -2 | 3,9 |
|  | Temporal Lobe, Middle Temporal Gyrus | 39 |  |  | -42 | -70 | 14 | 3,6 |
|  | Frontal Lobe, Precentral Gyrus | 6 | L | + | -42 | -16 | 62 | 3,9 |
|  |  | 4 |  |  | -39 | -28 | 66 | 3,5 |
| 28  *p<0.001 unc* | Frontal Lobe, Inferior Frontal Gyrus | 45 | L | + | -48 | 26 | 22 | 4,1 |
|  |  | 44 |  |  | -45 | 17 | 14 | 3,9 |
| 32  *p<0.001 unc* | Precuneus | 7 | R | + | 3 | -70 | 50 | 4,8 |
| 33  *p<0.05 FWE* | Frontal Lobe, Precentral Gyrus | 6 | R | + | 63 | 2 | 18 | Inf |
|  | Temporal Lobe, Superior Temporal Gyrus | 22 |  |  | 51 | -4 | 6 | Inf |
|  | Brain Stem | / |  | + | 6 | -25 | -34 | Inf |
|  | Frontal Lobe, Precentral Gyrus | 6 | L | + | -45 | -10 | 34 | Inf |
|  | Cerebellum, Posterior Lobe | / | R | - | 42 | -70 | -22 | Inf |
|  | Frontal Lobe, Middle Frontal Gyrus | 10 | L | - | -30 | 65 | 6 | Inf |
|  | Frontal Lobe, Superior Frontal Gyrus |  | R |  | 9 | 71 | 14 | 6,9 |
|  | Uncus | 20 | L | + | -24 | -4 | -38 | Inf |
|  | Frontal Lobe, Superior Frontal Gyrus | 8 | R | - | 33 | 32 | 50 | 7,6 |
|  | Frontal Lobe, Middle Frontal Gyrus | 6 | L |  | -33 | 5 | 58 | 7,4 |
|  | Occipital Lobe, Lingual Gyrus | 18 | R | - | 6 | -94 | -6 | 7,5 |
|  | Precuneus | 7 | R | - | 3 | -67 | 54 | 7,1 |
|  | Parietal Lobe, Superior Parietal Lobule |  |  |  | 12 | -73 | 62 | 6,1 |
|  | Cerebellum, Posterior Lobe | / | L | - | -48 | -67 | -30 | 6,9 |
|  | Parietal Lobe, Superior Parietal Lobule | 7 | L | - | -33 | -64 | 58 | 6,6 |
|  | Parietal Lobe, Inferior Parietal Lobule | 40 |  |  | -36 | -49 | 58 | 5,8 |
|  | Insula | 13 | R | + | 39 | -10 | 14 | 6,5 |
|  | Frontal Lobe, Middle Frontal Gyrus | 10 | R | - | 30 | 65 | 6 | 6,5 |
|  | Frontal Lobe, Superior Frontal Gyrus |  |  |  | 24 | 53 | 2 | 5,3 |
|  | Insula | 13 | L | + | -42 | 5 | -6 | 6,5 |
|  | Occipital Lobe, Cuneus | 19 | R | - | 30 | -88 | 22 | 6,5 |
|  | Precuneus |  |  |  | 36 | -79 | 34 | 6,1 |
|  | Temporal Lobe, Middle Temporal Gyrus | 38 | R | - | 48 | -1 | -42 | 6,1 |
|  | Frontal Lobe, Inferior Frontal Gyrus | 9 | R | + | 60 | 14 | 34 | 6 |
|  | Temporal Lobe, Middle Temporal Gyrus | 21 | R | - | 69 | -37 | -6 | 5,9 |
|  | Occipital Lobe, Middle Occipital Gyrus | 19 | L | - | -51 | -67 | -6 | 5,5 |
|  | Precuneus | 19 | L | - | -30 | -76 | 42 | 5,5 |
|  | Precuneus | 23 | L | - | 0 | -58 | 18 | 5,5 |
| 34  *p<0.05 FWE* | Cerebellum, Posterior Lobe | / | R | + | 42 | -64 | -22 | 5,2 |
|  | Cerebellum, Culmen |  |  |  | 42 | -49 | -18 | 5,2 |
|  | Occipital Lobe, Lingual Gyrus | 17 | L | + | -9 | -88 | 2 | 5,1 |
|  |  | 18 |  |  | -9 | -82 | -10 | 4,7 |
|  | Occipital Lobe, Fusiform Gyrus | 19 |  |  | -21 | -64 | -6 | 4,5 |
|  | Precuneus | 31 | R | + | 6 | -46 | 30 | 4,8 |
|  |  | 7 |  |  | 3 | -49 | 42 | 3,6 |
|  | Frontal Lobe, Precentral Gyrus | 6 | L | + | -63 | -16 | 38 | 4,6 |
|  | Parietal Lobe, Postcentral Gyrus | 1 |  |  | -57 | -25 | 38 | 3,6 |
|  | Frontal Lobe, Precentral Gyrus | 6 | R | + | 54 | -7 | 6 | 4,1 |
|  | Parietal Lobe, Postcentral Gyrus | 1 |  |  | 66 | -16 | 22 | 4 |
|  |  | 43 |  |  | 57 | -7 | 18 | 3,2 |
|  | Temporal Lobe, Superior Temporal Gyrus | 41 | R | + | 48 | -31 | 14 | 3,8 |
|  | Temporal Lobe, Middle Temporal Gyrus | 21 |  |  | 54 | -28 | 2 | 3,4 |
| 35  *p<0.001 unc* | Parietal Lobe, Postcentral Gyrus | 43 | L | + | -57 | -7 | 18 | 4,1 |
|  | Frontal Lobe, Precentral Gyrus | 6 |  |  | -42 | -13 | 34 | 3,8 |

**Figure 2s: EEG-fMRI group analysis in 25 patients with temporal lobe epilepsy.**

Results of the group analysis (cluster size threshold K =0).Clusters are shown in sagittal slices of an anatomical template found in xjview toolbox (<https://www.alivelearn.net/xjview>). Color bar represents t-values.

**
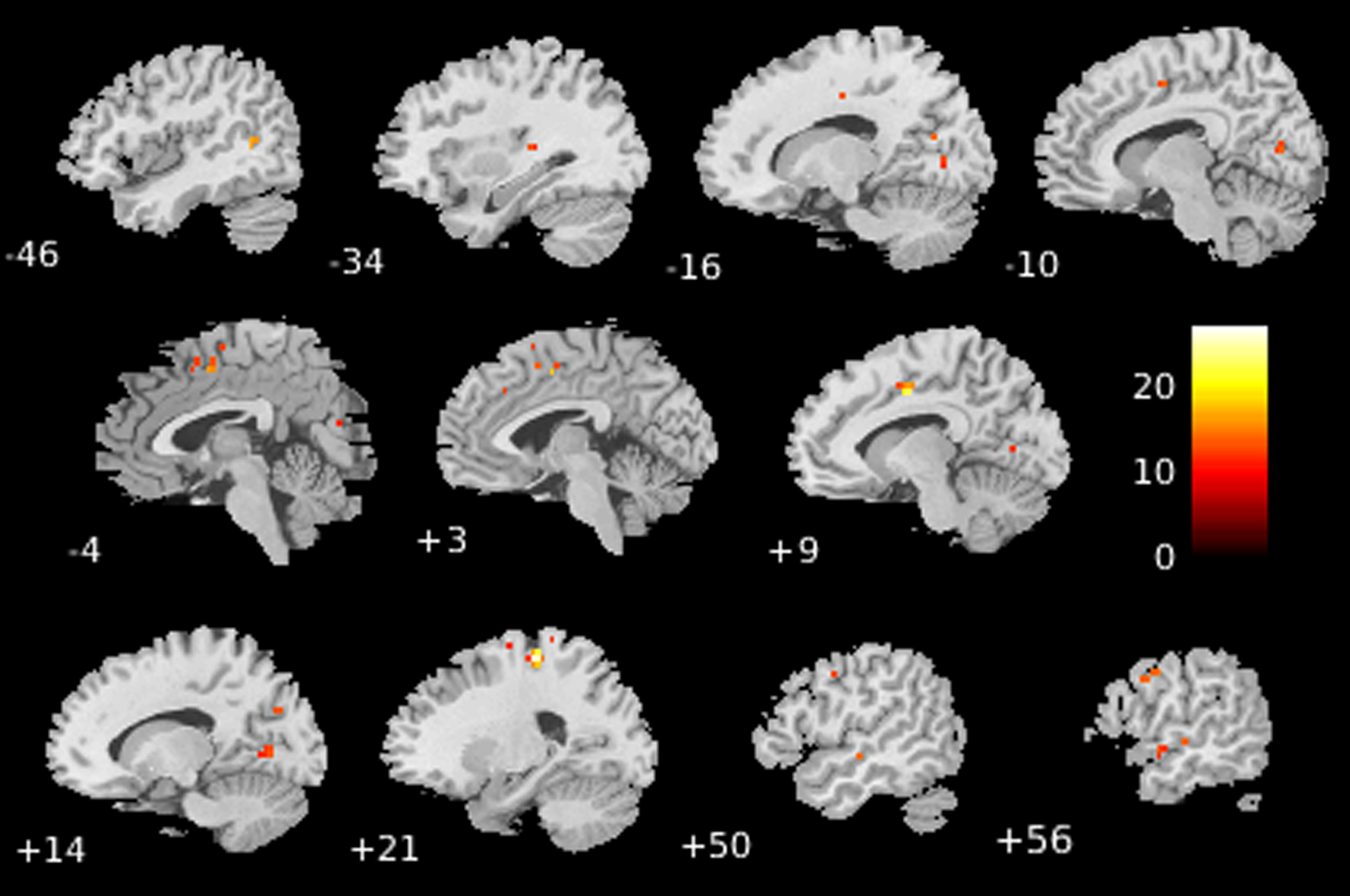
**
